# Quantifying the Leaping Motion Using a Self-Propelled Bionic Robotic Dolphin Platform

**DOI:** 10.3390/biomimetics8010021

**Published:** 2023-01-05

**Authors:** Junzhi Yu, Tianzhu Wang, Di Chen, Yan Meng

**Affiliations:** 1 State Key Laboratory of Management and Control for Complex Systems, Institute of Automation, Chinese Academy of Sciences, Beijing 100190, China; 2 State Key Laboratory for Turbulence and Complex Systems, Department of Advanced Manufacturing and Robotics, College of Engineering, Peking University, Beijing 100871, China; 3 College of Electronics and Information Engineering, Guangdong Ocean University, Zhanjiang 524088, China; 4 School of Artificial Intelligence, University of Chinese Academy of Sciences, Beijing 100049, China

**Keywords:** robotic dolphin, leaping motion, dynamic model, motion analysis

## Abstract

Kinematic analysis of leaping motions can provide meaningful insights into unraveling the efficient and agile propulsive mechanisms in dolphin swimming. However, undisturbed kinematic examination of live dolphins has been very scarce due to the restriction of close-up biological observation with a motion capture system. The main objective of this study is to quantify the leaping motion of a self-propelled bionic robotic dolphin using a combined numerical and experimental method. More specifically, a dynamic model was established for the hydrodynamic analysis of a changeable submerged portion, and experimental data were then employed to identify hydrodynamic parameters and validate the effectiveness. The effects of wave-making resistance were explored, indicating that there is a varying nonlinear relationship between power and speed at different depths. In addition, the wave-making resistance can be reduced significantly when swimming at a certain depth, which leads to a higher speed and less consumed power. Quantitative estimation of leaping motion is carried out, and the results suggest that with increase of the exiting velocity and angle, the maximum height of the center of mass (CM) increases as well; furthermore, a small exiting angle usually requires a much larger exiting velocity to achieve a complete exiting motion. These findings provide implications for optimizing motion performance, which is an integral part of underwater operations in complex aquatic environments.

## 1. Introduction

Unmanned vehicles, encompassing unmanned surface vehicles and unmanned underwater vehicles, have received increasing attention over the past several decades. They have found widespread applications in complex, hazardous, and even extreme aquatic environments, such as search and rescue [1], environmental survey and monitoring [2], underwater infrastructure inspection and maintenance [3], sea exploration [4], and oceanographic observation [5]. Although traditional propulsion technologies are well-developed and easy-to-use, underwater robots and vehicles powered by rotating propellers are often bulky, noisy, and not sufficiently maneuverable, which hardly meets the ambitious requirements of high maneuverability and stealthiness in marine areas. As is well-known, fish swim with small wakes and low disturbance. Therefore, bionic underwater robots borrowing structural and functional inspiration from aquatic organisms (e.g., fish and dolphins) are advantageous for better maneuverability, adaptability, and quietness in certain cases [6]. Much of the impetus of the growing interest in biomimetics comes from the appealing promise of being able to learn and utilize optimizations attained over millions of years, as current biological structures and functions are persistently optimized via evolution.

In nature, cetaceans are a mainly aquatic order of mammals including whales, dolphins, and porpoises. Remarkably, in comparison with propeller-driven underwater robots, dolphins can maneuver rapidly in tight space, accelerate and decelerate more swiftly, and even leap out of the water as high as 15 feet, relying on an integrated propulsion and steering system as a whole. In addition to biological relevance, as a typical complex system involving different disciplines, dolphin-inspired robotic research offers vital clues for next-generation innovative underwater robots. To analyse dolphin motions, Tanaka et al. measured the time-varying kinematics of a dolphin in an aquarium by recording the three-dimensional (3D) trajectories of burst-accelerating swimming [7]. Even though Mendelson et al. explored the water exit dynamics of archerfish with experimental measurements and numerical simulations [8,9]. Owing to the limitations of close-up biological observation and on-site motion capture, in reality it is not easy to implement undisturbed kinematic examination of live dolphins’ leaping motions. With the aid of a well-developed robotic dolphin platform, quantification of a dolphin’s leaping motion becomes possible. Thus, bionic robotic dolphins can serve as a useful and multipurpose platform for examining kinematics, dynamics, control, and extensive aquatic applications.

Up to now, the majority of dolphin-inspired robotic research has been concentrated on the development and verification of novel dolphin-like robots, ranging from mechanical design [10,11] to measurement and hydrodynamic analysis [12] and control methods [13]. The first proof-of-concept self-propelled dolphin robot can be traced back to the year of 1999 [14]. After that, Nakashima’s group developed a second generation of dolphin-like robot and investigated 3D maneuverability through the coordinated movements of caudal, dorsal, and pectoral fins [15]. Yu’s group concentrated on mechanism design and motion control of diverse robotic dolphins with the capability of dorsoventral swimming, gliding, and even leaping [16,17,18]. In most of the existing literature on dolphin-like swimming mimicry, unfortunately, the self-propelled leaping motion across the water–air interface is rarely replicated. It is generally acknowledged that achieving high speed is the first step towards crossing the water–air interface. Therefore, reducing drag as significantly as possible during the ignition and acceleration phases plays a critical role. When a body moves near the water surface at high speed, the wave-making resistance becomes the primary drag, consuming energy very evidently [19,20]. Extensive investigations on the propulsion efficiency of robotic fish, including system design and control optimization, were conducted in [21,22,23,24,25]. The influences of wave-making resistance on the propulsion performance of a bionic robotic dolphin has been less analyzed.

The leaping motion with water surface crossing is extremely complicated, as well as fascinating. Korobkin et al. investigated the hydrodynamic forces of a smooth elongated body during exit from the water surface and proposed a simplified model of water exiting motion [26]. Hu et al. established a motion model of the water-to-air process for an unmanned submersible aerial vehicle with a regular shape [27]. Moreover, the numerical method was used to validate the motion model. Chang et al. proposed a theoretical model to analyze the vertical height of the axisymmetric body, and a simple spring system used to shoot a body through the water surface was designed for model validation [28]. The results suggest that the entrained fluid plays a significant role in limiting the maximum jumping height. Jiang et al. developed a miniature water surface jumping robot. A carbon fiber strip was selected as the energy storage component to actuate two wings to flap the water surface, and a simplified mathematical model was built to predict the jumping performance [29]. In our previous work [16], a numerical model with the force equilibrium equations was proposed to estimate the minimum exit speed of a robotic dolphin. As mentioned above, these conducted analyses of leaping motion are mainly aimed at simple bodies with regular profiles and specified exiting speeds. The leaping motion for robots with complex shapes and self-propelled bodies is more comprehensive and needs further investigation.

In this paper, we mainly focus on quantifying the leaping motion using a self-propelled bionic robotic dolphin. To this end, aiming at a developed robotic dolphin, a dynamic model of leaping motion with dorsoventral propulsion is established. The Morrison equation is employed to analyze the time-variant hydrodynamic forces acting on the submerged body. In addition, in pursuit of high swimming performance, the effects of wave-making resistance on propulsion performance, including speed and power, are analyzed. The hydrodynamic parameters are identified through the swimming experiments, then a quantitative analysis of leaping motion is provided. This paper provides new insights into the design and control of a novel underwater robot with the capability of leaping motion.

The rest of this paper is organized as follows. Section 2 introduces the mechanical design and prototype of a robotic dolphin with the capability of leaping. The dynamic model of leaping motion with dorsoventral propulsion is presented in Section 3. Experiments and motion analyses are offered in Section 4. Finally, Section 5 provides the conclusions of this paper.

## 2. Overview of the Leaping Robotic Dolphin

**Figure 1:**
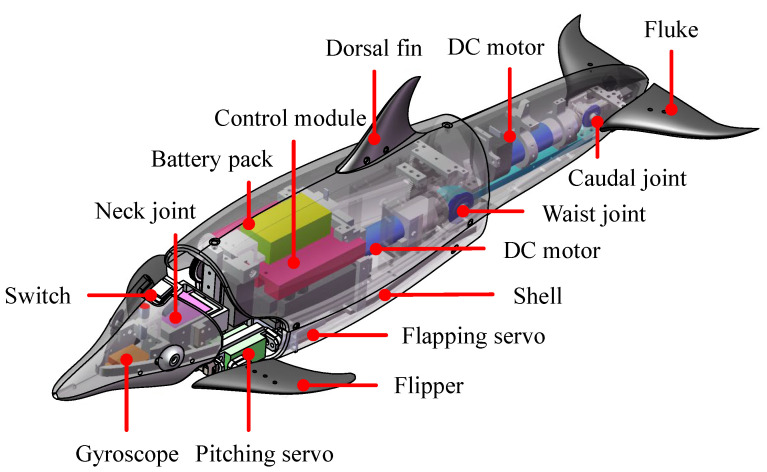
Mechanical design of the bionic robotic dolphin.

As illustrated in Figure 1, a robotic dolphin with the capability of leaping motion was developed in our previous work [16]. To reduce the hydrodynamic drag, a well-streamlined body profile drawing from a spotted dolphin was adopted. With dorsoventral oscillation, two joints, a waist joint and a tail joint, were configured in the posterior body to ensure powerful propulsion. A neck joint used exclusively for the head-lead nose-up and nose-down is integrated. In addition, a pair of two-degree-of-freedom flippers for realizing the flapping motion and feathering motion is implemented. Combining the flippers and two-joint propulsion body, the 3D swimming motion, including both pitch and yaw maneuverability, can be realized. However, to simplify the analysis of the robotic dolphin’s leaping motion, the flippers’ deflection angles are 0°. A pair of flukes attach to the caudal joint, producing the thrust of swimming. These fins are all made of polypropylene and designed with a NACA 0018 airfoil. Certain materials, such as titanium alloy, aluminum alloy, and nylon, were adopted to meet the requirements of light weight and high strength. A customized elastic skin with a thickness of 1 mm protects the robot from water.

**Figure 2:**
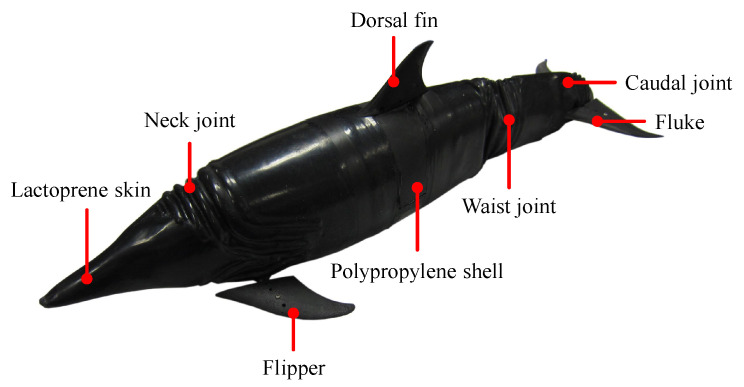
Prototype of the bionic robotic dolphin.

A variety of electronic components including sensors, control units, communication modules, and power supply, are imported into the robotic dolphin. A miniature attitude heading reference system (AHRS, MicroStrain, 3DM-GX3-25) is mounted in the head to provide the attitude data of the robotic dolphin. A pressure sensor (SQsensor, CYG-515A) is utilized to measure the depth information. A control board with various communication interfaces implements the control algorithm and external orders. In addition, dedicated motor controllers (MAXON, EPOS2 50/5) are employed to obtain the state information of the joints, such as their speed and position. With the integration of mechanical structure and electronic modules, the prototype is fabricated with a length of 0.72 m and a mass of 4.7 kg, as shown in Figure 2. The detailed parameters are tabulated in Table 1.

## 3. Dynamic Modeling of Leaping Motion

The complete process of the leaping motion ordinarily contains three phases [17]. (1) The water-exiting phase: beginning at the penetration of the water surface until achieving complete separation between the flukes and the water surface. During this phase, the robotic dolphin oscillates its body and flukes continuously to provide thrust. (2) The projectile phase: the robotic dolphin enters the air completely and is only dominated by gravity. (3) The water reentry phase: starting from when the tip of the nose touches the water surface until the flukes are submerged in the water. During the water reentry phase, the body and flukes of the robotic dolphin remain still, and its dynamic model including the hydrodynamic analysis and buoyancy analysis is similar to that of the water-exiting phase; therefore, in this paper we primarily concentrate on the dynamic model of the water-exiting phase.

### 3.1. Kinematic Analysis

**Figure 3:**
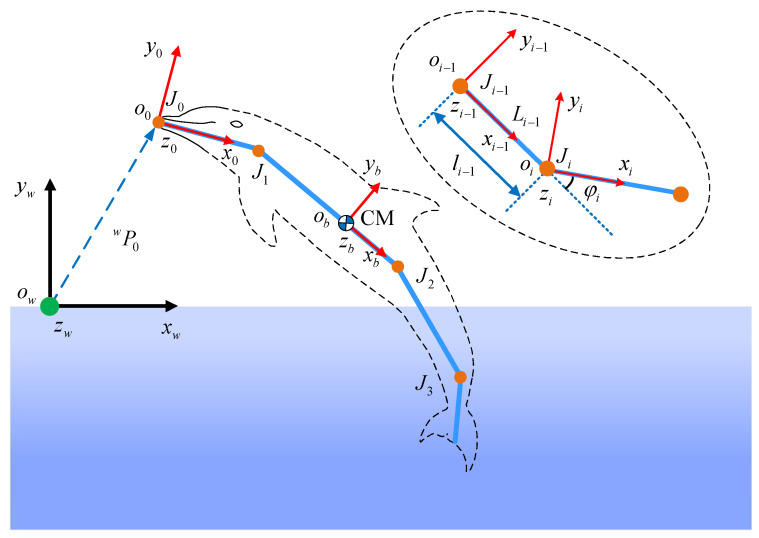
Illustration of coordination frames and notations.

To analyze the cross-domain locomotion of a robotic dolphin, a dynamic model was built in detail. As shown in Figure 3, coordinate frames and notations are defined to describe the dynamic model clearly. The robotic dolphin can be simplified as a four-actuated-link structure in series. The inertial coordinate frame is defined as $C_{w}=o_{w}-x_{w}y_{w}z_{w}$ and the relative coordinate frames are denoted as $C_{i}=o_{i}-x_{i}y_{i}z_{i},\left(i=0,1,2,3\right)$. In particular, a body coordinate frame $C_{b}=o_{b}-x_{b}y_{b}z_{b}$ which relates to the center of mass (CM) is defined. For the CM of the dolphin located at $L_{1}$, therefore, $C_{b}$ is parallel to $C_{1}$. All of the coordinate frames conform to the right-hand rule. The plane $x_{w}o_{w}y_{w}$ is parallel to the vertical plane. The origin $o_{i},\left(i=0,1,2,3\right)$ located at the joint $J_{i},\left(i=0,1,2,3\right)$ and axis $o_{i}x_{i}$ is overlapped with link $L_{i}$ and points to $J_{i+1}$. Here, $J_{0}$ indicates the tip point of the robotic dolphin’s head, while $J_{1}$, $J_{2}$, and $J_{3}$ denote the neck joint, waist joint, and caudal joint, respectively. To simplify the dynamic model, in this paper only the leaping motion in the vertical plane is considered. The length of link $L_{i}$ is denoted as $l_{i}$, while $\varphi_{i}$ indicates the joint angle of $J_{i},\left(i=1,2,3\right)$ and $\theta_{0}$ represents the pitch angle of the robotic dolphin. It should be noted that the head of the robotic dolphin, with neck joint $J_{1}$, is mainly designed to provide the pitch moment for posture adjustment in the air, which is not considered in this paper. Therefore, the neck joint $J_{1}$ is locked in the water-exiting process.

The kinematics of the robotic dolphin can be derived using a similar method to that for robotic fishes [30,31]. The rotation matrix of frame $C_{i}$ with respect to $C_{w}$ can be provided as follows:

(1) $${}^{w}R_{i}=\left(\begin{array}{ccc} \cos\theta_{i} & -\sin\theta_{i} & 0\\ \sin\theta_{i} & \cos\theta_{i} & 0\\ 0 & 0 & 1 \end{array}\right),$$

where $\theta_{i}=\theta_{0}+\sum_{j=1}^{i}\varphi_{j},\left(i=1,2,3\right)$ represents the angle between axis $o_{i}x_{i}$ and axis $o_{w}x_{w}$.

Let $r_{i}\left(l\right)$ represents the position vector of an arbitrary point in the *i*th link; it can be calculated as the position vector sum between $J_{i}$ and point of link $L_{i}$ in frame $C_{w}$:

(2) $$r_{i}\left(l\right)={}^{w}P_{i}+{}^{w}R_{i}P_{i}\left(l\right),\left(i=0,1,2,3\right)$$

where

(3) $${}^{w}P_{i}={}^{w}P_{0}+\sum_{j=1}^{i}{}^{w}R_{j-1}{}^{j-1}P_{j},\left(i=1,2,3\right)\;\mathrm{and}\;P_{i}\left(l\right)={\left[l,0,0\right]}^{T}$$

where ${}^{w}P_{0}$ is the position vector of $J_{0}$ in frame $C_{w}$, ${}^{j-1}P_{j}={\left[l_{i-1},0,0\right]}^{T}$ denotes the position vector of $J_{j}$ in frame $C_{j-1}$, and *l* denotes the distance of the arbitrary point in *i*th link to joint $J_{i}$.

The translational velocity and angular velocity can be obtained by taking the time derivative of the position vector and angular vector in $C_{w}$, and can be formalized through the coordinate transformation as follows:

(4) $$\left\{\begin{array}{c} {}^{w}V_{i}={\dot{r}}_{i}={}^{w}R_{i}{}^{i}V_{i}\\ {}^{w}\Omega_{i}={}^{w}\Omega_{0}+{}^{w}R_{i}{}^{i}\Omega_{i},\left(i=1,2,3\right). \end{array}\right.$$

where ${}^{w}\Omega_{0}={\left[0,0,\dot{\theta_{0}}\right]}^{T}$ is the angular velocity vector of link $L_{0}$.

The acceleration can be further obtained by taking the time derivative of the velocity as follows:

(5) $$\left\{\begin{array}{c} {}^{w}{\dot{V}}_{i}={}^{w}R_{i}{}^{i}{\dot{V}}_{i}+{}^{w}R_{i}{}^{w}{\hat{\Omega }}_{i}{}^{i}V_{i}\\ {}^{w}{\dot{\Omega }}_{i}={}^{w}{\dot{\Omega }}_{0}+{}^{w}R_{i}{}^{i}{\dot{\Omega }}_{i},\left(i=1,2,3\right), \end{array}\right.$$

where ${}^{w}{\dot{R}}_{i}={}^{w}R_{i}{}^{w}{\hat{\Omega }}_{i}$ and ${}^{w}{\hat{\Omega }}_{i}$ is the skew-symmetric matrix of angular velocity vector ${}^{w}\Omega_{i}$.

The velocity and acceleration of an arbitrary point in these links can be obtained with the derivation of the provided kinematic and actuation control of the joints.

### 3.2. Hydrodynamic Analysis

During the leaping motion, two fluids with strikingly different physical properties are involved. When considering the density of air and the exiting velocity of a robotic dolphin, the aerodynamic forces are neglected. Therefore, only hydrodynamic forces are calculated. It should be noted that the interaction between the robot and surrounding fluid is extremely complicated; in addition, with the increase in leaping height, the length of a body immersed in water varies, which brings about changes in buoyancy and hydrodynamic forces. As only vertical motion is of concern here, the rolling inertia and yaw inertia are not considered in the hydrodynamic analysis, nor is the entrained fluid caused by the exited body is not.

**Figure 4:**
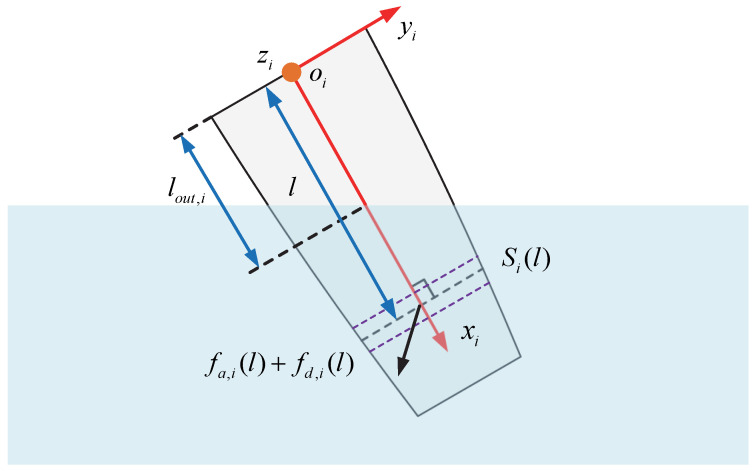
Illustration of the hydrodynamic forces exerted on *i*th link $L_{i}$ with an exiting length of $l_{out,i}$.

In this paper, the Morrison equation is employed to analyze the hydrodynamic forces acting on the body, which include the added mass force and the drag force [32]. Note that only the remaining immersed section is subject to hydrodynamic forces. Figure 4 illustrates the hydrodynamic forces acting on the *i*th link which is crossing the water surface with an exiting length of $l_{out,i}$. For the per unit length of the body with a distance of *l* to $J_{i}$, the hydrodynamic forces are considered to act on the cross-section $S_{i}\left(l\right)$. The added mass forces are due to the response of the surrounding water, which is accelerated by the oscillation of the robotic dolphin. To account for the decrease in the submerged length of each link, the added mass in the longitudinal and transverse direction of the link are all considered. For simplicity, we directly calculate the added mass force expressed in inertial frame $C_{w}$ below:

(6) $$f_{a,i}\left(l\right)=-m_{a,i}\left(l\right){\ddot{r}}_{i}\left(l\right),m_{a,i}=\frac{1}{4}c_{m,i}\rho \pi {h_{i}\left(l\right)}^{2},$$

where $m_{a,i}\left(l\right)$ is the added mass of $S_{i}\left(l\right)$, $c_{m,i}$ indicates the dimensionless coefficient, ρ denotes the density of the fluid, and $h_{i}\left(l\right)$ denotes the immersed height. By integrating $f_{a,i}\left(l\right)$ along axis $x_{i}$, the added mass force acting on the link $L_{i}$ can be calculated.

The drag force generated by the fluid slice in a cross-section $S_{i}\left(l\right)$ can be obtained as follows:

(7) $${}^{i}f_{d,i}\left(l\right)=-\frac{1}{2}\left[\begin{array}{c} c_{f,i}\rho p_{i}\left(l\right)\left|v_{x,i}\left(l\right)\right|v_{x,i}\left(l\right)\\ c_{d,i}\rho h_{i}\left(l\right)\left|v_{y,i}\left(l\right)\right|v_{y,i}\left(l\right)\\ 0 \end{array}\right],$$

where $c_{f,i}$ and $c_{d,i}$ are the dimensionless hydrodynamic coefficients, $p_{i}\left(l\right)$ denotes the perimeter of the cross-section $S_{i}\left(l\right)$, and $v_{x,i}\left(l\right)$ and $v_{y,i}\left(l\right)$ are the velocity in the direction of axes $x_{i}$ and $y_{i}$, respectively.

As shown in Figure 4, the hydrodynamic forces exerted on the immersed segment of link $L_{i}$ can be derived by integrating the forces acting on cross-section $S_{i}\left(l\right)$ along axis $x_{i}$ as follows:

(8) $$F_{i}=\int_{l_{out,i}}^{l_{i}}\left(f_{a,i}\left(l\right)+f_{d,i}\left(l\right)\right)dl$$

where $f_{d,i}\left(l\right)$ means the drag force expressed in inertial frame $C_{w}$.

During the leaping motion, the submerged length of link $L_{i}$ is changing along with time, that is, the integrating range needs to be updated in each step time. For the link which is totally beneath the water surface, the exiting length $l_{out,i}$ is zero.

The hydrodynamic forces for a fluke designed with an airfoil profile for high-speed swimming are calculated with the lift and drag model, as follows:

(9) $$\left\{\begin{array}{c} F_{L}=\frac{1}{2}\rho C_{l}\left(\alpha \right)V_{c}^{2}S_{c}\\ F_{D}=\frac{1}{2}\rho C_{d}\left(\alpha \right)V_{c}^{2}S_{c} \end{array},\right.$$

where α is the angle of attack, $C_{l}$ and $C_{d}$ are the dimensionless lift coefficient and drag coefficient, respectively, $V_{c}$ is the linear velocity magnitude of flukes, and $S_{c}$ means the wetted area of flukes.

The moment $\tau_{i}$ generated by the hydrodynamic force $F_{i}$ acting on the CM of the robotic dolphin can be calculated as follows:

(10) $$\tau_{i}=\left(r_{i}-r_{g}\right)\times F_{i},i=0,1,2,3,$$

where $r_{g}$ indicates the position vector of CM in inertial frame $C_{w}$.

### 3.3. Buoyancy Analysis

**Figure 5:**
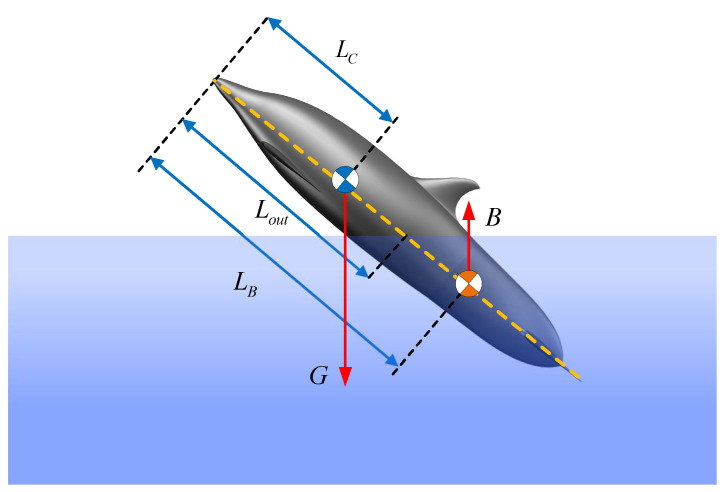
Illustration of the variations in the submerged volume and position of the center of buoyancy.

**Figure 6:**
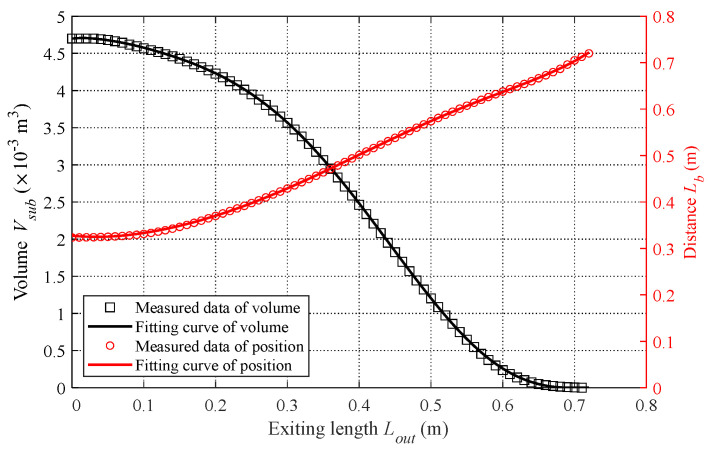
Volume and position of the CB of the submerged body under different exiting lengths.

In addition to the analyzed hydrodynamic forces, the robotic dolphin is affected by the forces of gravity and buoyancy, as illustrated in Figure 5. To simplify the analysis, the positions of CM and CB are defined as $L_{C}=0.323$ m and $L_{B}$, which indicate the respective distances to the CM and CB from the top point of the robot along the longitudinal axis of the body. During the leaping motion of the robotic dolphin, the volume of the submerged portion $V_{\left(sub\right)}$ and the location of the submerged body’s center of buoyancy (CB) vary with the exiting length $L_{out}$, which is defined as the distance between head to the water surface along the body. Considering the irregular shape of the robotic dolphin, it is difficult to describe the changes in volume and CB position accurately. Therefore, we assume that the slight variation in the position of the CM and CB during oscillation of the robotic dolphin can be ignored. In addition, the density of the robotic dolphin is considered to be the same as that of water. Taking advantage of SolidWorks, the volume and location of the CB under different exiting lengths $L_{out}$ are measured, then the fitting method is utilized to acquire the function of $V_{sub}\left(L_{out}\right)$ and $L_{b}\left(L_{out}\right)$, as shown in Figure 6. The change in the buoyancy force and moment with the exiting length $L_{out}$ can be provided as follows:

(11) $$B=\left(\begin{array}{c} 0\\ \rho gV_{sub}\left(L_{out}\right)\\ 0 \end{array}\right),\tau_{B}=\left(L_{b}\left(L_{out}\right)-L_{c}\right)Bcos\theta_{0}$$

Finally according to the analysis mentioned above, the dynamic model takes the following form:

(12) V˙Ω˙=M−1Fh+B+Gτh+τB

where M is the total inertia matrix, including the inertial matrix and added mass matrix, $F_{h}=\sum_{i=0}^{3}F_{i},\left(i=0,1,2,3\right)$ is the total hydrodynamic force acting on each link, and $\tau_{h}=\sum_{i=0}^{3}\tau_{i},\left(i=0,1,2,3\right)$ is the total torque generated by the fluid relative to the CM.

## 4. Experiments and Model Analysis

### 4.1. Swimming Speed and Power Testing

**Figure 7:**
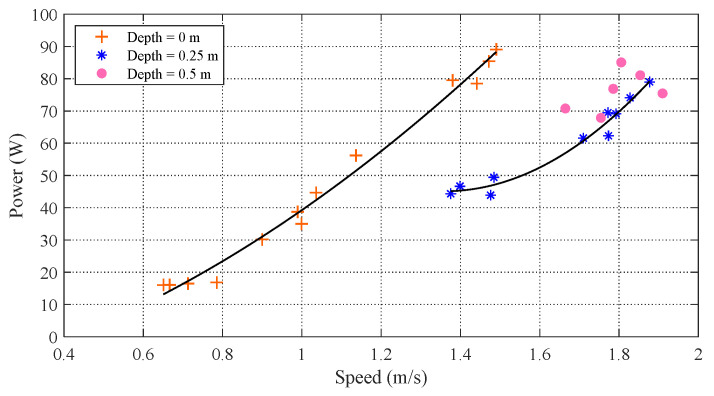
Comparison of power at different swimming depths.

Achieving high speed is essential for crossing the water-air interface. Therefore, reducing drag in the ignition and acceleration phases plays a critical role. When a body such as a boat moves near the water surface, the surrounding fluid is pushed away and waves are created, which produces drag, that is, wave-making resistance. In particular, the wave-making resistance rises sharply with the increase in speed, which causes a great deal of energy dissipation. In this paper, in order to pursue high swimming speed and efficiency, the effects of wave-making resistance on a robotic dolphin are explored. Extensive experiments with different control parameters were conducted to measure the swimming performance, including speed and power, at three different depths (0 m, 0.25 m, and 0.5 m) below the water surface. As presented in Figure 7, for the most part the power increases with increasing speed. In particular, at the same speed, the power at a depth of 0.25 m is much less than that of the robotic dolphin when swimming at the surface. For example, at the water surface (depth = 0 m), the maximum speed is about 1.49 m/s (2.07 BL/s), corresponding to a power of 89.06 W. With a similar speed (1.48 m/s, 2.06 BL/s), the consumed power at a depth of 0.25 m is only about 49.44 W. The cost of transport (COT), denoting the consumed energy per unit distance traveled, improves by 44.5%. With increasing depth (depth = 0.5 m) the differences of power at the same speed are not obvious, indicating that the influence of wave-making resistance is significantly reduced. During the acceleration phase before leaping, swimming at a depth larger than 0.25 m contributes to achieving both a higher swimming speed and greater efficiency.

The fitting method is used here to describe the relationship between speed and power at depths of 0 m and 0.25 m, with the respective results as follows:

(13) $$\left\{\begin{array}{c} P_{1}=30.0911U^{2}+25.1135U-15.9416\\ P_{2}=127.3315U^{2}-346.2896U+280.5573 \end{array}.\right.$$

**Figure 8:**
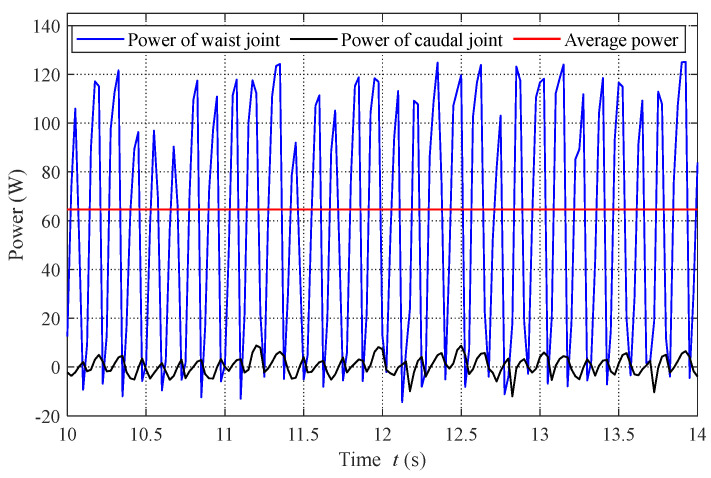
Measured power of the robotic dolphin during swimming.

These aquatic experiments indicate that there is a varying nonlinear relationship between power and speed at different depths. With increasing speed, much more power is needed to overcome the drag produced by the fluid. Moreover, in our experiments, a maximum speed of 1.9 m/s (2.65 BL/s) was achieved at a depth of 0.5 m. Figure 8 provides the power curves of the waist joint and caudal joint during the swimming motion as recorded by the motor controller. The oscillation frequencies of both joints are set to 4 Hz. It can be seen that the consumed power of the waist joint is much larger than that of the caudal joint. The drag is mainly overcome by the waist joint, a determination that can provide guidance for optimizing robotic dolphin design. During the swimming motion at a constant frequency, the average power of the robotic dolphin is calculated with the average of instantaneous power from time 10 s to 14 s. For clarity, a red curve indicating a constant value of 64.6 W is presented in Figure 8.

### 4.2. Leaping Motion Analysis

#### 4.2.1. Parameters Identification

**Figure 9:**
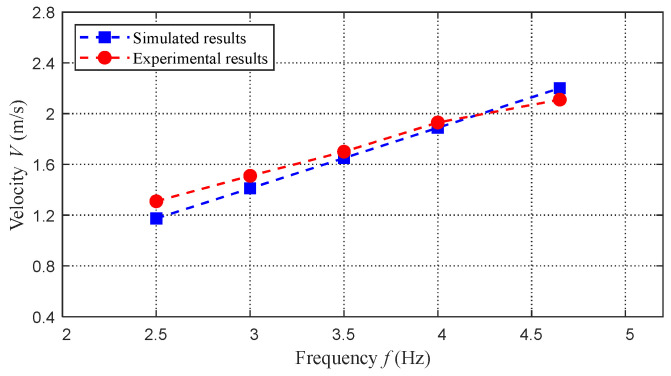
Comparisons between simulated results and experimental results.

The derived dynamic model involves numerous physical parameters and hydrodynamic parameters. By taking advantage of the mechanical model in SolidWorks, we can obtain the physical parameters easily, as tabulated in Table 2. As for the hydrodynamic model, the swimming speed is crucial for the leaping motion; therefore, the measured speed of the robotic dolphin in [17] is utilized to identify these hydrodynamic parameters. The swimming motion can be taken as a special situation of the established dynamic model. Concretely, during the swimming motion, the gravity and buoyancy counteract each other, and the length $l_{out,i},\left(i=0,1,2,3\right)$ is zero. Finally, the obtained hydrodynamic parameters are tabulated in Table 3. By integrating these obtained parameters into the dynamic model, the swimming speeds at different frequencies were simulated and compared with experiment results, as presented in Figure 9. It can be seen that these results match well with each other, validating the effectiveness of the built dynamic model at least to an extent.

#### 4.2.2. Water-Exiting Phase Analysis

**Figure 10:**
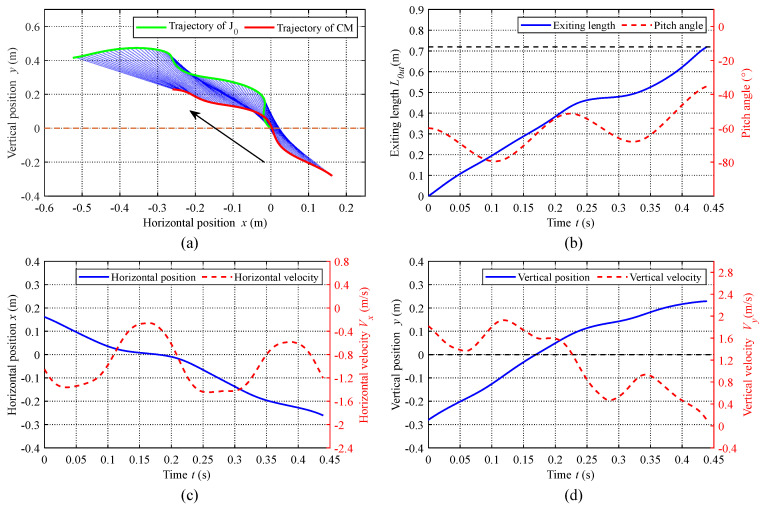
Curves of state variables during water-exiting phase with $V_{e}=2.1$ m/s and $\theta_{e}=-60^{\circ }$. (**a**) Trajectories of $J_{0}$ (green curve) and the CM (red curve). (**b**) Pitch angle and exiting length of the robotic dolphin. (**c**) Position and velocity in the horizontal direction. (**d**) Position and velocity in the vertical direction.

In this section, we use the validated dynamic model to estimate the states of leaping motions quantificationally by numerical simulations. The water-exiting motion ends when the robotic dolphin exits the water completely ($L_{out}=0.72$ m) or the velocity in the vertical direction $V_{y}$ is zero. In the simulation, an initial position of $J_{0}$ is given at the water surface ${\left(0,0,0\right)}^{T}$; then, the water-exiting motion can be analysed by presetting different exiting velocities corresponding to different oscillation frequencies and exiting angles. To further validate the built dynamic model, we estimate the maximum leaping height of CM with an exit angle of 54° and exit velocity of 1.93 m/s as 15.6 cm. Compared with the experimental result of 17.5 cm [17], the relative error is about 10.9%. Therefore, we can utilize the final obtained dynamic model to estimate the leaping motion states. As an illustration, we set the initial exit velocity and angle as $V_{e}=2.1$ m/s and $\theta_{e}=-60^{\circ }$, respectively, and evaluated the motion states during the water-exiting phase. Figure 10a presents the trajectories of $J_{0}$ (green curve) and the CM (red curve). The arrow shows the direction of the exiting motion. The starting points of $J_{0}$ and CM are located at positions (0,0) and (0.1105,−0.3035), respectively. The blue line connects $J_{0}$ and CM at the same moment, which can reflect the variation of the robotic dolphin’s posture intuitively. Concretely, Figure 10b shows the pitch angle and exiting length of the robotic dolphin. It can be seen that the variation of pitch angle is undulant and the absolute value decreases gradually. The exiting length $L_{out}$ increases to the length of the robotic dolphin (0.72 m), indicating the end of the water-exiting phase. The positions and velocities in the horizontal and vertical directions are shown in Figure 10c,d, respectively. For the high-frequency oscillation of the body, the velocities in both directions have the characteristic of variation in undulation. For the horizontal drag this is small; while the horizontal velocity varies a great deal, this decreases slightly in undulation. In addition, due to the gradual increase in vertical drag, the vertical velocity varies, and the main tendency is a rapid decline. Specifically, it drops from 1.819 m/s to 0.101 m/s. As mentioned above, the feature of undulation in the curves of the states is mainly attributed to the high-frequency oscillation propulsion of body.

**Figure 11:**
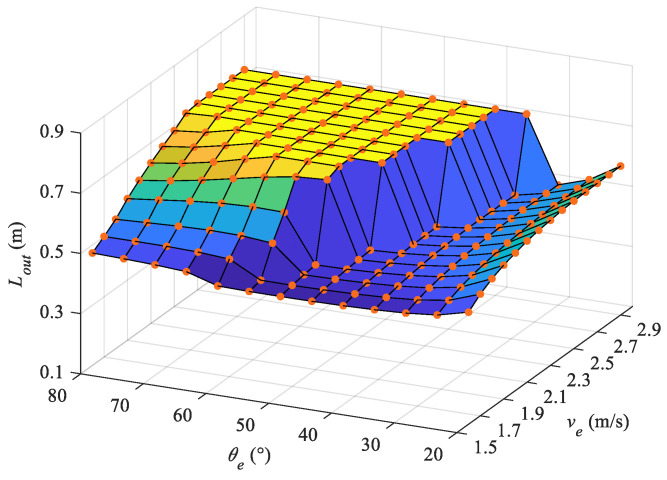
Exiting lengths at different exit velocities and angles.

To analyze the influence of the exit velocity $V_{e}$ and angle $\theta_{e}$ on the leaping motion, we can estimate the exiting length $L_{out}$ with the built dynamic model. A velocity from 1.6 m/s to 2.9 m/s with an interval of 0.1 m/s and angle in the range of $\left[20^{\circ },80^{\circ }\right]$ with an interval of $5^{\circ }$ are selected in the simulation. The results are shown in Figure 11. It should be noted that when the leaping length $L_{out}=0.72$ m, the water-exiting phase ends and the simulation stops, as illustrated in the plane section with maximum height. At the same exit angle, with the rise of exit velocity $V_{e}$, the exiting length $L_{out}$ increases as well. In particular, at a high exit angle $\theta_{e}$ (in the range of $\left[50^{\circ },80^{\circ }\right]$) the exiting length increases more apparently until achieving the complete exiting motion. In addition, with the same exiting velocity $V_{e}$, the minimum $L_{out}$ is usually obtained at the middle exiting angle, which is close to the angle achieving complete water-exiting motion. At a lower angle $\theta_{e}$, even if the vertical velocity is small, which limits the motion time, the large horizontal velocity contributes to a higher exiting length.

**Figure 12:**
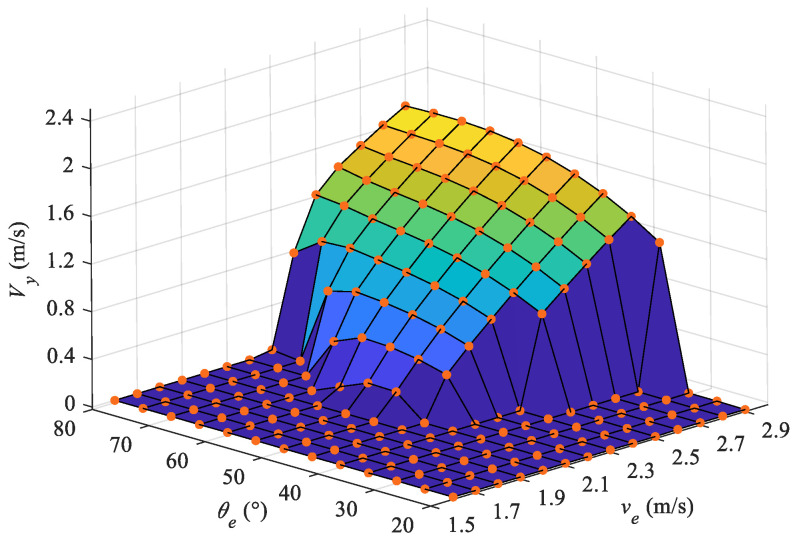
Velocities at the end of the water-exiting phase.

Figure 12 shows the vertical velocity at the end of the water-exiting phase, from which we can intuitively observe whether the robot exits completely. The values which are equal to zero represent incomplete water-exiting motions. At the velocity of 2.1 m/s, the exit angles of $50^{\circ }$, $55^{\circ }$, and $60^{\circ }$ are the first group of angles that realize the complete water-exiting motion. With increasing velocity, larger exit angles make it easier to exit the water completely. For example, at a velocity of 2.4 m/s with an exit angle of $80^{\circ }$, the robotic dolphin has leaped out of water completely, however, for the situation with an exiting angle of $35^{\circ }$, the robot requires a velocity greater than 2.8 m/s to exit the water. From Figure 11 and Figure 12, it can be seen that the exiting length is extremely sensitive to the exit angle near the minimum exit angle at each exit velocity. There are two reasons accounting for this phenomenon. The leaping length $L_{out}=y_{0}csc\left(\theta_{0}\right)$ is determined by the vertical position of $J_{0}$ and the pitch angle $\theta_{0}$. For the low ebb in Figure 11, when the exiting velocity is low, the vertical components are small, and the smaller exiting angle with a large value of cosec function trends towards obtaining a larger exiting length. However, with increasing velocity the larger exit angle leads to an increase in the vertical velocity component, resulting in a higher exiting time, and in turn greater exiting length. Thus, even if the robotic dolphin requires a larger velocity to exit the water completely at a low exit angle, it can obtain a greater exiting length at lower exit velocities. Figure 10 suggests that the pitch angle decreases in undulation. The pitch angle at the exiting moment is very important for the calculation of $L_{out}$. At a large velocity with high oscillation frequency, the robotic dolphin is more sensitive to the pitch angle.

#### 4.2.3. Projectile Phase Analysis

The projectile phase under the effect of gravity alone that follows the complete water-exiting phase ($L_{out}=0.72$ m) is analyzed as well. The horizontal, vertical, and rotation motions can be described as follows:

(14) $$\left\{\begin{array}{c} {\dot{x}}_{g}={\dot{x}}_{g}\left(t_{1}\right)\\ {\dot{y}}_{g}=-g\left(t-t_{1}\right)+{\dot{y}}_{g}\left(t_{1}\right)\\ {\dot{\theta }}_{0}={\dot{\theta }}_{0}\left(t_{1}\right) \end{array},t\in \left(t_{1},t_{2}\right],\right.$$

(15) $$\left\{\begin{array}{c} x_{g}={\dot{x}}_{g}\left(t_{1}\right)t+x_{g}\left(t_{1}\right)\\ y_{g}=-\frac{1}{2}g{\left(t-t_{1}\right)}^{2}+{\dot{y}}_{g}\left(t_{1}\right)\left(t-t_{1}\right)+y_{g}\left(t_{1}\right)\\ \theta_{0}={\dot{\theta }}_{0}\left(t_{1}\right)\left(t-t_{1}\right)+\theta_{0}\left(t_{1}\right) \end{array},t\in \left(t_{1},t_{2}\right],\right.$$

where $t_{1}$ means the end time of the water-exiting phase, $x_{g}\left(t_{1}\right)$, $y_{g}\left(t_{1}\right)$, $\theta_{0}\left(t_{1}\right)$, ${\dot{x}}_{g}\left(t_{1}\right)$, ${\dot{y}}_{g}\left(t_{1}\right)$, and ${\dot{\theta }}_{0}\left(t_{1}\right)$ indicate the end states of the water-exiting phase, and $t_{2}$ is the end time of a projectile phase when the tip point of the robotic dolphin touches the water surface.

**Figure 13:**
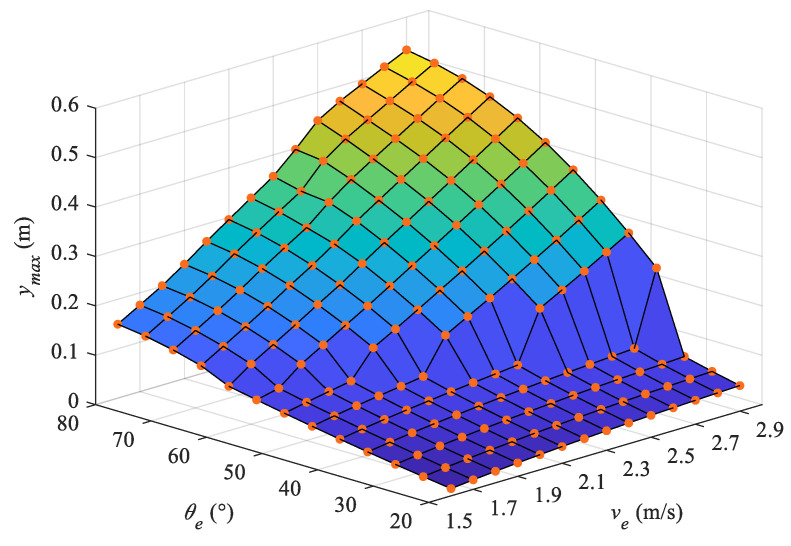
Maximum height of center of mass in the leaping motion.

Utilizing the terminative states of the complete water-exiting phase, we further analyze the motion in the projectile phase. The maximum heights of the robotic dolphin’s CM at different exiting states are calculated and presented in Figure 13. For the situation of an incomplete water-exiting motion, the maximum height is obtained at the end of the water-exiting phase ($v_{y}=0$ m/s). It is apparent that the leaping height increases gradually with increasing exit velocity. Furthermore, the leaping height increases with the rising exit angle. However, for exit angles that achieve an exit velocity, there is a sharp increase of leaping height, and the exit angle gradually decreases with the increase in exit velocity. Comparing Figure 11 and Figure 13, it can be seen that the exiting length is quite different from the leaping height, which is mainly due to the influence of the pitch angle.

## 5. Discussion

In this paper, we have analyzed the leaping motion with the built dynamic model. In nature, the leaping motion for a robotic dolphin includes two modes, namely, exiting out of the water surface straightly or with posture adjustment. The first mode considered in this paper requires higher velocity to realize the complete water-exiting motion, and usually pursues the leaping height. Concerning the second mode of the robotic dolphin, achieving complete separation from the water surface requires a lower leaping height, which is easier to achieve and ordinarily pursues the leaping distance. The conducted experiments in our previous study achieved the second mode; therefore, in the present research, we mainly focus on the mode involving a straight exiting from the water surface.

In dynamic modeling, many assumptions have been made to simplify the hydrodynamic analysis. In [33,34,35], the robotic fish body or tail is taken as a slender body or flat plates. Therefore, the added mass coefficient in the longitudinal axis is usually very small and can be neglected, and the added mass force is calculated with the only the transverse acceleration. For our robotic dolphin, the length $l_{i}$ of each link is larger than the body height $h_{i}$ with a limiting ratio of $l_{i}/h_{i}$. In addition, during the leaping motion, the submerged length of each link gradually decreases with time, causing a decreasing ratio. Thus, we did not ignore the added mass in the direction of each link’s longitudinal axis. In [21], the added mass in the longitudinal axis of body was considered. Therefore, we directly calculate the added mass force expressed in the inertial frame with the absolute acceleration. When the robotic dolphin moves in the fluid with the oscillation of body, the surrounding water is accelerated in the two axial directions of the inertial frame. Then, we calculate the added mass forces produced by the reaction of the surrounding fluid with the added mass and accelerations. In order to improve the accuracy of our simplified dynamic model, we identify these hydrodynamic parameters with experimental swimming speed, which reshapes the dynamic model with data-driven feature. Even though many simplifications have been made in our hydrodynamic model, the experimental results in swimming speed and leaping height demonstrate its effectiveness.

Unlike our previous analysis of the water-exiting motion, the self-propelled propulsion at different velocities corresponds to different oscillation frequencies of the body, causing the leaping motion to be more sensitive to the pitch angle. For smaller exit angles, the vertical velocity component limits the maximum height of the CM, causing extremely short leaping times and making it more difficult to exit the water completely. In our experiments, the effects of the wave-making resistance are only explored with respect to the speed and power, which are essential for the leaping motion. In future work, further theoretical analysis needs to be conducted. Moreover, further optimization of the design and control system needs to be considered in order to achieve better leaping motion performance.

## 6. Conclusions

In this paper, we have presented a dynamic model for a self-propelled robotic dolphin in order to quantify its leaping motion. To analyze the hydrodynamic forces during the crossing of water surface, the Morrison equation with varying integral lengths is proposed. To account for the irregular shape of the robotic dolphin, the fitting method is utilized to describe the volume and position of the buoyancy center for the submerged portion, which vary with time. Experimental data are employed to identify the hydrodynamic parameters as well as to validate the established dynamic model. The influence of the wave-making resistance on speed and power is analyzed. The results suggest that when the robotic dolphin swims at a depth of greater than 25 cm, there is an obvious decrease in the wave-making resistance, which helps it to achieve a higher speed while consuming less power. Finally, the effects of the exit velocity and angle on the leaping motion, including the exiting length and maximum height, are analyzed. The obtained results indicate that the exit velocity and angle play a significant role in the leaping motion; with a small exit angle, the robotic dolphin has more difficulty achieving the complete exit motion.

## Figures and Tables

**Table 1:** Technical specifications of the robotic dolphin prototype.

Items	Characteristics
Mass	4.7 kg
Dimension (L × W × H )	0.72 m × 0.12 m × 0.13 m
Joint configuration	Body joint × 3, pectoral fin joint × 4
Controller	STM32F103ZET6 (ARM Cortex-M3)
Motor	DC motor × 2, servo motor × 5
Sensor	AHRS, pressure sensor
Power supply	29.6 V rechargeable batteries
Communication unit	Wireless (RF200, 433 MHz)

**Table 2:** Configuration parameters of the robotic dolphin.

Items	Unit	$L_{0}$	$L_{1}$	$L_{2}$	$L_{3}$
$m_{i}$	kg	0.692	3.124	0.727	0.157
$l_{i}$	m	0.174	0.278	0.158	0.11
$l_{c,i}$	m	0.128	0.132	0.065	0.033
$I_{r,i}$	kg·m${}^{2}$ ($\times 10^{-1}$)	1.983	2.436	1.896	1.865
$S_{c}$	m${}^{2}$ ($\times 10^{-2}$)	-	-	-	1.676

**Table 3:** Hydrodynamic parameters of the robotic dolphin.

Parameters	$c_{m,0}$	$c_{m,1}$	$c_{m,2}$	$c_{f,0}$	$c_{f,1}$	$c_{f,2}$	$c_{d,0}$	$c_{d,1}$	$c_{d,2}$
Value	0.25	0.24	0.20	0.006	0.008	0.004	0.45	1.25	0.4

## Data Availability

The data generated during the current study are available from the corresponding author on reasonable request.
